# Seoul National University Renal Stone Complexity Score for Predicting Stone-Free Rate after Percutaneous Nephrolithotomy

**DOI:** 10.1371/journal.pone.0065888

**Published:** 2013-06-18

**Authors:** Chang Wook Jeong, Jin-Woo Jung, Woo Heon Cha, Byung Ki Lee, Sangchul Lee, Seong Jin Jeong, Sung Kyu Hong, Seok-Soo Byun, Sang Eun Lee

**Affiliations:** 1 Department of Urology, Seoul National University Bundang Hospital, Seongnam, Korea; 2 Department of Urology, College of Medicine, Seoul National University, Seoul, Korea; University of KwaZulu-Natal, South Africa

## Abstract

**Objectives:**

Currently, no standardized method is available to predict success rate after percutaneous nephrolithotomy. We devised and validated the Seoul National University Renal Stone Complexity (S-ReSC) scoring system for predicting the stone-free rate after single-tract percutaneous nephrolithotomy (sPCNL).

**Patients and Methods:**

The data of 155 consecutive patients who underwent sPCNL were retrospectively analyzed. Preoperative computed tomography images were reviewed. The S-ReSC score was assigned from 1 to 9 based on the number of sites involved in the renal pelvis (#1), superior and inferior major calyceal groups (#2–3), and anterior and posterior minor calyceal groups of the superior (#4–5), middle (#6–7), and inferior calyx (#8–9). The inter- and intra-observer agreements were accessed using the weighted kappa (κ). The stone-free rate and complication rate were evaluated according to the S-ReSC score. The predictive accuracy of the S-ReSC score was assessed using the area under the receiver operating characteristic curve (AUC).

**Results:**

The overall SFR was 72.3%. The mean S-ReSC score was 3.15±2.1. The weighted kappas for the inter- and intra-observer agreements were 0.832 and 0.982, respectively. The SFRs in low (1 and 2), medium (3 and 4), and high (5 or higher) S-ReSC scores were 96.0%, 69.0%, and 28.9%, respectively (p<0.001). The predictive accuracy was very high (AUC 0.860). After adjusting for other variables, the S-ReSC score was still a significant predictor of the SFR by multiple logistic regression. The complication rates were increased to low (18.7%), medium (28.6%), and high (34.2%) (p = 0.166).

**Conclusions:**

The S-ReSC scoring system is easy to use and reproducible. This score accurately predicts the stone-free rate after sPCNL. Furthermore, this score represents the complexity of surgery.

## Introduction

The incidence of urolithiasis is approximately 5% to 10% in the general population, and 30% of the surgical workload is related to urinary stone treatment in an active urologic department [1], [2]. Large renal stones can cause pain, hematuria, infection, renal function deterioration, and mortality [3]. Thus, large renal stones should be actively treated, except in patients who are otherwise too ill to tolerate stone removal. Among several treatment options, percutaneous nephrolithotomy (PCNL) is a standard treatment for patients with large renal stones [3], [4], because it has been demonstrated to have the highest stone-free rate (SFR) [3]. However, it does not result in a 100% SFR. Furthermore, many patients experience significant complications despite its minimally invasive nature. Overall, the recently published Clinical Research Office of the Endourological Society (CROES) PCNL global study reported an SFR of 75.5% and a complication rate of 20.5% [5]. The clinical results vary widely case by case because of the varying characteristics of renal stones. A higher stone burden and complexity is associated with a lower SFR and a higher complication rate [3], [6]. There have been some proposed models to predict the results for renal stones [7], [8], however, none of them are accepted as the standardized method to predict the SFR after PCNL, yet. Thus, many urologists find it difficult to predict precise outcomes and tailor informed decision making with patients.

Our experience performing PCNL led to the hypothesis that the distributional complexity of stones is more associated with the SFR than the number or size of stones. Thus, we devised the Seoul National University Renal Stone Complexity (S-ReSC) scoring system to predict the SFR after PCNL. This scoring system is simply calculated by counting the number of sites involved among 9 pre-determined locations in the pelvicalyceal system. We evaluated the ability of the S-ReSC scoring system to predict the SFR and represent the surgical difficulty of single-tract PCNL (sPCNL).

## Materials and Methods

### Ethics Statement

This study was approved by the Institutional Review Board (IRB) of Seoul National University Bundang Hospital (Seongnam, Republic of Korea). The approval number is B-1211/178-109. We were given exemption from getting informed consents by the IRB. It was a retrospective study. And personal identifiers were all removed and the data were anonymously analyzed.

### Patients and Procedures

A total of 155 consecutive sPCNL cases were included from January 2004 through July 2012 at Seoul National University Bundang Hospital. Percutaneous renal access was routinely obtained by 2 experienced uro-radiologists 1 day before or on the operative day. If the patient previously had a percutaneous nephrostomy, this nephrostomy was used as an access. sPCNL was performed in a prone position by 1 of 4 faculty professors. A rigid nephroscope was used in combination with a ballistic lithotripter, stone forceps, and a suction tube. If needed, a flexible nephroscope and/or ureteroscope were also used for collecting systems that were inaccessible with a rigid nephroscope. In this setting, a Holmium laser and stone basket were used. Temporary drainage was usually maintained with a 14-F nephrostomy catheter.

All patients were evaluated with pre- and post-operative computed tomography (CT). The evaluated preoperative stone parameters included the number, largest diameter, total stone volume, renometry (complete, partial staghorn, or other), average Hounsfield units, and degree of hydronephrosis (normal, mild, moderate, or severe). A complete staghorn stone was defined as a renal pelvic calculi extending into all major calyceal groups filling at least 80% of the renal collecting system, and a partial staghorn stone was defined as a renal pelvic calculi extending into at least two calyceal groups. Stone volume was calculated by length×width×depth×π×0.52. The total stone volume was the sum of all stone volumes. The average Hounsfield unit was measured using the elliptical region of interest incorporated into the largest stone area in a non-contrast axial image [9]. “Stone-free” was defined as no evidence of residual stones on postoperative images for 1 month.

### S-ReSC Scoring

**Figure 1:**
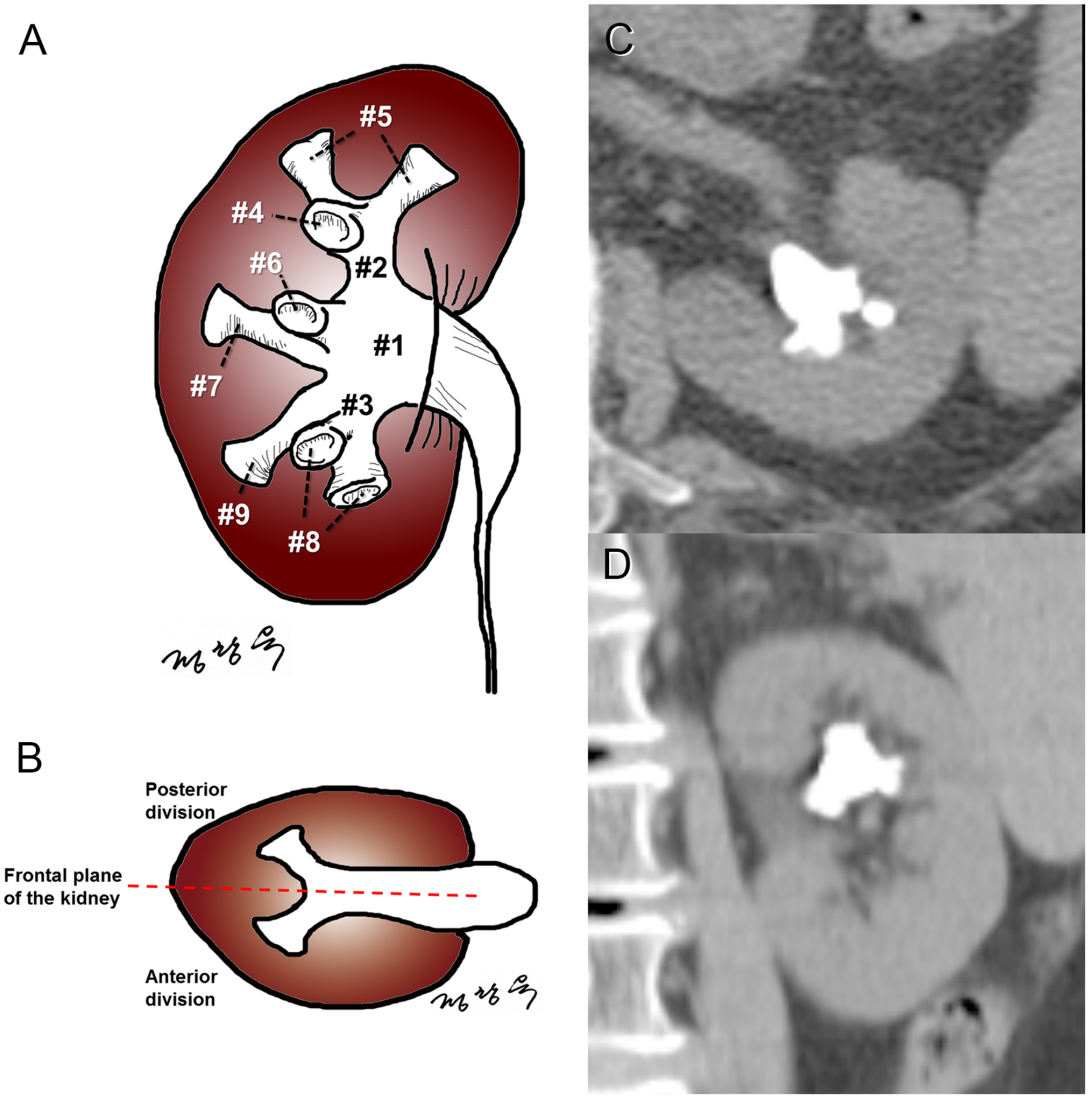
Schematic representation of the Seoul National University Renal Stone Complexity (S-ReSC) scoring system. The score is calculated by counting the number of sites involved, regardless of the size and number of the stones. (a) The sites were as follows; the renal pelvis (#1), the superior and inferior major calyceal groups (major calyx and infundibulum) (#2–3), and the anterior and posterior minor calyceal groups of the superior (#4–5), middle (#6–7), and inferior calyx (#8–9). (b) The anterior and posterior division was simply divided using the frontal plane of the kidney. Example of computed tomography axial (c) and coronal (d) images of the renal stone with S-ReSC score 4. The renal stone involved the renal pelvis (#1), superior major calyceal group (#2), and the anterior and posterior minor calyceal groups of the superior calyx (#4 and #5).

The S-ReSC score was calculated by counting the number of sites involved, regardless of the size and number of stones. The sites were as follows; the renal pelvis (#1), superior and inferior major calyceal groups (major calyx and infundibulum) (#2–3), and anterior and posterior minor calyceal groups of the superior (#4–5), middle (#6–7), and inferior calyx (#8–9). The anterior and posterior division was simply divided using the frontal plane of the kidney. Each minor calyceal group could have more than one calyx. Minor anatomical variants in the minor calyceal system were simplified to provide simple and quick scoring and augment reproducibility. One point was given for each location, resulting in a maximum of 9 points in total (Fig. 1).

Univariate logistic regression analyses were performed to verify the effect of stone existence at each site.

### Evaluation of Reproducibility

Initially, 1 junior faculty member appraised the S-ReSC score. This score set was used for the other statistical analyses. To test intra-observer agreement, this faculty member rescored the patients 1 month later and was blinded to the first reading. To assess inter-observer agreement, one senior resident independently scored the S-ReSC. Inter- and intra-observer agreements were conservatively evaluated using the weighted kappa (κ) with the squared method.

### Validation as a Predictive Method

The SFRs were examined according to the S-ReSC scores. To evaluate the predictive accuracy, the area under the receiver operating characteristic curve (AUC) was used. Next, the predicted probability versus the actual SFR was compared. This examination was performed by calibration plotting using 200 bootstrap resamples. To test the clinical utility of the S-ReSC score, a decision curve analysis (DCA) was constructed. The AUC value only presents the predictive accuracy, while a DCA depicts the potential net benefit of the predictive model at each threshold probability using a graph [10]–[12].

We also performed univariate logistic regression analyses to predict the stone-free. The assessed variables were the S-ReSC score, age, sex, laterality, body mass index, previous treatment, stone number (1, 2, 3 or >3), largest diameter, total stone volume, renometry, average Hounsfield units, and degree of hydronephrosis. To adjust for other variables, the S-ReSC score and all significant variables (p<0.05) were included in a multiple logistic regression analysis.

To evaluate the correlation between the S-ReSC score and the difficulty and complexity of the surgery, we compared the operative time, hemoglobin drop (preoperative hemoglobin – immediate postoperative hemoglobin), and complication rate. Complications were assessed and scored according to the modified Clavien classification [13], [14].

The data are expressed as the mean ± standard deviation of the mean. To compare mean values, one-way analysis of variance (ANOVA) test was used. When other statistical test was used, we indicated the method with the results. A p-value <0.05 was considered to be statistically significant. All statistical analyses were performed using R for Windows, version 2.15.2 (http://www.r-progect.org/). Calibration plot and decision curve were also generated using this software package.

## Results

The basic characteristics of the patients are shown in Table 1. The overall SFR was 72.3% (112/155). Additional treatments were used due to residual stones in 11.6% of patients (18/155; shockwave lithotripsy in 10, retrograde intrarenal surgery in 8, and secondary PCNL in 2), and the final stone-free was achieved in 76.8% (119/155).

**Table 1:** Patient demographics and basic renal stone characteristics.

Variables	
No. of patients	155
Age, year, mean ± SD	54.9±13.6
Body mass index, Kg/m^2^, mean ± SD	25.5±3.6
Gender, no. (%)	
Male	101 (65.2)
Female	54 (34.8)
Previous treatment, no. (%)	
Shock wave lithotripsy	35 (22.6)
Retrograde intrarenal surgery	5 (3.2)
Percutaneous nephrolithotomy	12 (7.7)
Laterality, no. (%)	
Right	76 (49.0)
Left	79 (51.0)
Number of stone, no. (%)	
1	88 (56.8)
2	28 (18.1)
3	8 (5.2)
>3	31 (20.0)
The largest diameter, mm, mean ± SD	23.6±9.2
Total stone volume, cm^3^, mean ± SD	13.5±21.9
Average Houndsfield unit, mean ± SD	820.7±336.8
Renometry	
Complete staghorn	19 (12.3)
Partial staghorn	29 (18.7)
Renal pelvis+multiple calyces	23 (14.8)
Renal pelvis+single calyx	26 (16.8)
Renal pelvis only	38 (24.5)
Multiple calyces	14 (9.0)
Single calyx	6 (3.9)
Degree of hydronephrosis, no. (%)	
Normal	26 (16.8)
Mild	58 (37.4)
Moderate	42 (27.1)
Severe	29 (18.7)
Major stone composition, no. (%)	
Calcium oxalate monohydrate	75 (48.4)
Calcium oxalate dehydrate	21 (13.5)
Calcium phosphate	5 (3.2)
Carbonite apatite	7 (4.5)
Uric acid	23 (14.8)
Struvite	9 (5.8)
Cystine	2 (1.3)
Others	11 (7.1)
Missing	2 (1.3)

The mean S-ReSC score was 3.15±2.12. The S-ReSC scores of complete (7.16±1.36) and partial staghorn stones (4.17±1.14) were significantly higher than the other stone types (2.17±1.31, p<0.001). The effect of stone existence in the anterior calyces on the SFR was similar to posterior calyces (Table 2).

**Table 2:** The odds ratios by stone location for estimating the stone-free (univariate regression analyses).

Stone location	Odds ratio	95% Confidence interval	P value
Renal pelvis	0.502	0.160–1.572	0.237
Upper major calyx	0.357	0.153–0.833	0.017
Lower major calyx	0.302	0.142–0.641	0.002
Upper anterior minor calyx	0.140	0.041–0.483	0.002
Upper posterior minor calyx	0.226	0.087–0.585	0.002
Mid anterior minor calyx	0.281	0.113–0.700	0.006
Mid posterior minor calyx	0.199	0.075–0.530	0.001
Lower anterior minor calyx	0.214	0.101–0.453	<0.001
Lower posterior minor calyx	0.224	0.106–0.472	<0.001

The weighted kappas for inter- and intra-observer agreement were 0.832 (95% confidence interval [CI] 0.764–0.900) and 0.982 (95% CI 0.971–0.992), respectively, indicating that the scale could be interpreted as having “very good reliability”. The senior resident (2.79±2.02) tended to score lower than the junior faculty member (paired samples t-test, p<0.001). However, the intra-observer agreement was almost perfect. Major disagreement occurred for S-ReSC scores of 4 or higher.

**Figure 2:**
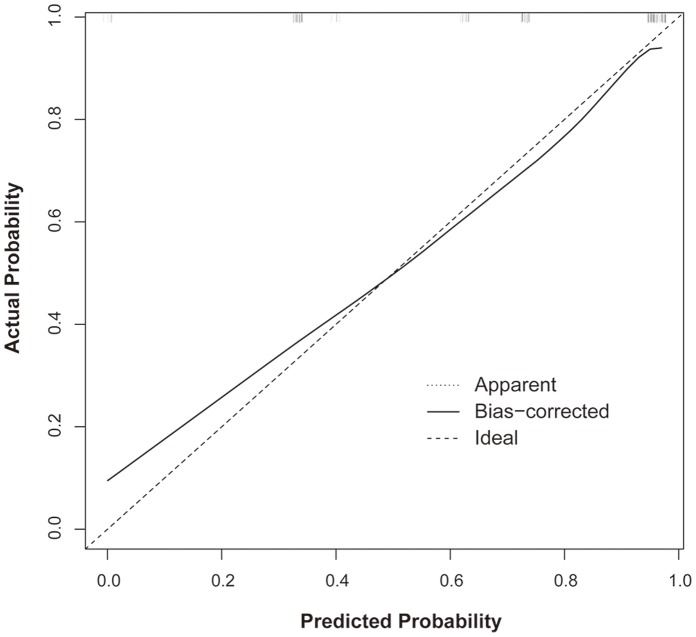
A calibration plot of the Seoul National University Renal Stone Complexity (S-ReSC) score to predict the stone-free rate after single-tract percutaneous nephrolithotomy.

**Figure 3:**
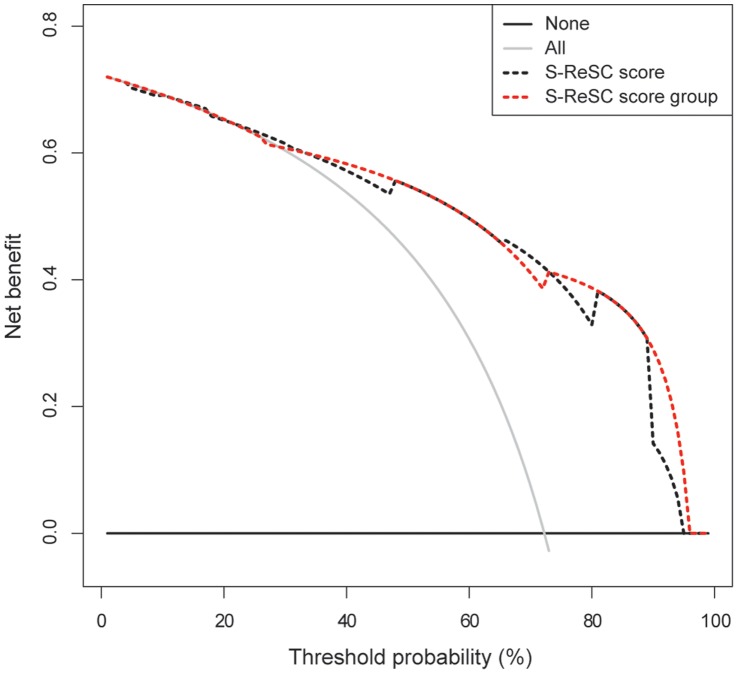
Decision curve analysis. The curve demonstrates a positive net benefit in almost all threshold probabilities using the Seoul National University Renal Stone Complexity (S-ReSC) score to predict the stone-free rate after single-tract percutaneous nephrolithotomy.

The SFRs according to the S-ReSC scores are shown in Table 3. The scores could be roughly grouped into low (1–2), medium (3–4), and high (5–9) score groups. The SFRs were significantly decreased in the order of the low (96.0%), medium (69.0%), and high (28.9%) score groups (chi-square test, p<0.001). The AUCs of the original and 3-tiered S-ReSC score groups were 0.860 (95% CI 0.793–0.927) and 0.853 (95% CI 0.787–0.919), respectively. The calibration plot showed a well-calibrated prediction that had little over- or under-estimation (mean absolute error 0.027) (Fig. 2). The DCA demonstrated a positive net benefit for almost all threshold probabilities in the original and 3-tiered S-ReSC score groups (Fig. 3).

**Table 3:** The stone-free rates according to the Seoul National University Renal Stone Complexity (S-ReSC) score and the S-ReSC score group.

S-ReSC score	Stone-free rate	S-ReSC score group	Stone-free rate
1	95.2% (40/42)	Low (1–2)	96.0% (72/75)
2	97.0% (32/33)		
3	73.1% (19/26)	Medium (3–4)	69.0% (29/42)
4	62.5% (10/16)		
5	33.3% (5/15)	High (5–9)	28.9% (11/38)
6	33.3% (3/9)		
7	40.0% (2/5)		
8	0% (0/6)		
9	33.3% (1/3)		
P<0.001		P<0.001	

The S-ReSC score, stone number, largest diameter, total stone volume, renometry, average Hounsfield units, and degree of hydronephrosis were significantly associated with the stone-free. Even after adjusting for all other significant variables, the S-ReSC score was strongly correlated with the stone-free after sPCNL (Table 4).

**Table 4:** Logistic regression analyses to predict the stone-free after single-tract percutaneous nephrolithotomy.

Variables	Unadjusted		Adjusted (original score)		Adjusted (score group)	
	Odds ratio	95% CI	Odds ratio	95% CI	Odds ratio	95% CI
S-ReSC score	0.477	0.373–0.610	0.310	0.180–0.532		
S-ReSC score group						
Medium vs. low	0.093	0.025–0.351			0.026	0.004–0.159
High vs. low	0.017	0.004–0.066			0.003	0.001–0.041
Stone number						
2 vs. 1	0.616	0.222–1.710	1.026	0.263–4.001	1.332	0.293–6.064
3 vs. 1	0.205	0.046–0.915	0.510	0.044–5.892	0.921	0.035–24.169
>3 vs. 1	0.169	0.069–0.416	0.885	0.210–3.735	1.327	0.301–5.856
Largest diameter	0.946	0.909–0.983	1.002	0.902–1.113	1.001	0.903–1.109
Total stone volume	0.981	0.963–1.000	1.029	0.985–1.074	1.015	0.976–1.055
Average Hounsfield unit	0.998	0.997–1.000	0.999	0.997–1.000	0.999	0.997–1.000
Renometry						
Partial staghorn vs. others	0.767	0.289–2.035	4.676	0.966–22.630	7.455	1.482–37.514
Complete staghorn vs. others	0.065	0.020–0.217	4.581	0.454–46.208	1.691	0.183–15.627
Hydronephrosis						
Mild vs. none	3.367	1.309–8.660	0.184	0.029–1.156	0.257	0.047–1.408
Moderate vs. none	3.019	1.109–8.223	0.210	0.030–1.464	0.325	0.052–2.051
Severe vs. none	8.214	2.013–33.525	0.048	0.006–0.391	0.042	0.005–0.363

The higher S-ReSC score group had a significantly longer operative time (128.3±72.1 min. vs. 153.9±78.0 min. vs. 173.4±88.6 min, p = 0.013) and larger hemoglobin drop (0.9±1.2 mg/dL vs. 1.5±1.3 mg/dL vs. 1.7±1.8 mg/dL, p = 0.003). The complication rates showed an increasing trend in order to S-ReSC score groups (1.87% [14/75] vs. 28.6% [12/42] vs. 34.2% [13/38], respectively; p = 0.166). A total of 53 complications occurred in 39 (25.2%) patients. Most complications were mild and transient, but 7 (4.5%) and 4 (2.6%) patients experienced grade IIIA and IIIB complications, respectively (Table 5).

**Table 5:** Complications according to the modified Clavien classification.

Clavien grade	Complication	No.	%
I		13	8.4%
	Transient increase in creatinine >1.4 ng/dL (preoperatively creatinine ≤1.4 ng/dL)	6	3.9%
	Prolonged tract leakage	7	4.5%
II		29	18.7%
	Transfusion	16	10.3%
	Fever >38.0 with antibiotics	13	8.4%
IIIA		7	4.5%
	Sepsis	1	0.6%
	Nephrostomy tube insertion	2	1.3%
	Bleeding managed by angioembolization (under local anesthesia)	3	1.9%
	wound dehiscence	1	0.6%
IIIB		4	2.6%
	Open conversion	2	1.3%
	Ureteric stricture managed by ureterotomy or balloon dilation under general anesthesia	2	1.3%
Total		53 in 39 patients	34.2% in 25.2%

## Discussion

Understanding the urinary collecting system is very important to reliably perform endourologic procedures and uro-radiologic evaluations. Sampaio and colleges described the pelvicalyceal morphologic classification in an analysis of 140 3-dimensional polyester resin corrosion endocasts obtained from 70 fresh cadavers, and they proposed 2 major groups [15]. Group A (62.2%) contained the superior and inferior major calyceal groups and the mid-minor calyces drain of the two major calyxes. Group B (37.8%) comprised the pelvicalyceal systems with mid-calyceal drainage independent of both the superior and inferior calyceal groups. Group B was divided into 2 types according to the presence (Type B-I, 21.4%) or absence (Type B-II, 16.4%) of the mid-major calyx. Thus, only 21.4% of the kidney contains the mid-major calyx. Furthermore, the mid-major calyx typically has no or a very short infundibulum, and access to the mid-major calyx is not difficult. Therefore, we decided not to incorporate the mid-major calyx into the S-ReSC scoring system. The number of minor calyces varies among individuals, from 5 to 14. For simplicity and global application, we just classified 6 minor calyceal groups; the superior, mid, and inferior calyx groups with anterior and posterior divisions.

If a surgeon can access a certain pelvicalyceal site, they may be able to remove nearly all of the stones, regardless of their number or size in that location. The number of stones in different sites is clearly associated with the S-ReSC score. The stone burden is commonly expressed as the largest diameter or the estimated total stone volume [16], [17]. However, these methods are potentially inaccurate because of the complex shape of large renal stones [18]. To improve accuracy, computer software should be used for volumetric assessment, and mathematical correction methods should be applied [7], [18], [19]. Partial or complete staghorn stones suggest higher stone burden and complexity. Staghorn stones can be indicated using S-ReSC scores of 3 to 9. The S-ReSC score was significantly associated with this renometry, and it also described the complexity more precisely. Commonly accepted predictive factors, such as stone number, burden, and renometry, were closely associated with the S-ReSC score. Furthermore, the S-ReSC score could predict the SFR more accurately than these commonly accepted predictive factors. Indeed, the predictive accuracy of the S-ReSC score for the SFR after sPCNL was very high, with an AUC of 0.860. Additionally, the traditional predictive factors were overwhelmed by the S-ReSC score by interaction in a multiple regression analysis.

Several groups have attempted to identify significant predictors of the stone-free after PCNL [20], [21], and they have suggested stone size, number, location, and pelvicalyceal system anatomy as predictors. However, a significant predictor is itself not a predictive tool, and some predictors, such as the pelvicalyceal system anatomy, are very difficult and time consuming to measure. Recently, other research groups have reported the development of prediction models for PCNL [7], [8]. However, the proposed models have many pitfalls, thus they are not widely used. For example, ‘Guy’s stone score’ comprises 4 grades [8]. The grades were divided by stone number, location, simple versus abnormal anatomy, and staghorn stone status. The SFRs for grades 1 to 4 were 81%, 72.4%, 35%, and 29%, respectively. Meanwhile, they did not present the predictive accuracy or a proper validation process. Furthermore, the grading system was too narrative and not quantitative and was therefore not widely adopted. ‘Staghorn morphometry’ is a new clinical classification and predictive model for PCNL [7], [22]. Stone volume and collecting system anatomy have been assessed using 3-dimensional volume-rendering software based on CT urography. The classification of staghorn stones has been proposed as follows: type 1, total stone volume <5,000 mm^3^ and <5% of stones in unfavorable calyces; type 2, between type 1 and 3; and type 3, total stone volume >20,000 mm^3^ and >10% of stones in unfavorable calyces. An unfavorable calyx was defined as a calyx with an acute angle (no specific cut-off point was given) and an infundibular width of less than 8 mm. This classification is based on an exact measurement, but it is so complex and requires special software. Therefore, it is difficult to use in daily practice in most urological clinics. By contrast, the S-ReSC scoring system does not require additional software and is easy to use, requiring approximately 15 seconds to score in our experience.

The ideal prediction model in medicine should be generally accurate, well calibrated, simple, reproducible, and clinically meaningful [12], [23]–[25]. The S-ReSC scoring system has been evaluated with well-conducted validation efforts, and the results are presented here as the standard methodology [24], [25]. We are cautiously confident that the results are close to an ideal prediction model. In addition, the S-ReSC scoring system can be used as a method to define the complexity of a renal stone or surgery. It can be incorporated into the PCNL report as an overall index of complexity and can also be used in the case presentation as the representative index of complexity.

There are several limitations to the present study. This was a single-center study. Additionally, this is only a proposal of a predictive method for the SFR after PCNL and a descriptive method for presenting the complexity of renal stones. Thus, a larger scale multi-center study should be performed to validate our results. In our institution, experienced uro-radiologists obtain percutaneous renal access. If an urologist accesses the kidney, the stone-free probability and complication rate could be different. Additionally, if multi-tract or modified PCNL, such as supine PCNL, is performed, the results of the scoring system should be first confirmed.

In summary, the S-ReSC scoring system is easy to use and reproducible. This score accurately predicts the SFR after sPCNL. Furthermore, this score represents the complexity of surgery. Thus, the S-ReSC scoring system can be used as a predictive method to estimate the SFR after sPCNL and as a method for describing the complexity of renal stones.
